# Proposed Treatise on Typhoid and Typhus Fever

**Published:** 1847-12

**Authors:** 


### PROPOSED TREATISE ON TYPHOID AND TYPHUS FEVER.

Our friend Dr. Sutton, of Georgetown, Kentucky, as we learn, has it in contemplation to publish a historical and practical treatise on these fevers, as they have prevailed in the interior of this State ; and, especially, n Scott and the adjoining counties, where he has seen and treated them ; and to some extent observed their pathological anatomy. Dr. Sutton is already favorably known to our readers, by his contributions to this Journal. We cannot doubt that his proposed work will be a valuable contribution to our indigenous literature, and hope soon to hear of its being committed to the press.

